# Epidermal Growth Factor Receptor: A Potential Therapeutic Target for Diabetic Kidney Disease

**DOI:** 10.3389/fphar.2020.598910

**Published:** 2021-01-26

**Authors:** Lili Sheng, George Bayliss, Shougang Zhuang

**Affiliations:** ^1^ Department of Nephrology, Shanghai East Hospital, Tongji University School of Medicine, Shanghai, China; ^2^ Department of Medicine, Rhode Island Hospital and Alpert Medical School, Brown University, Providence, RI, United States

**Keywords:** Epidermal growth factor receptor, diabetic nephropathy, hemodynamic alternation, metabolic disturbance, inflammation, multicellular dysfunction

## Abstract

Diabetic kidney disease (DKD) is a leading cause of end-stage renal disease worldwide and the major cause of renal failure among patients on hemodialysis. Numerous studies have demonstrated that transient activation of epidermal growth factor receptor (EGFR) pathway is required for promoting kidney recovery from acute injury whereas its persistent activation is involved in the progression of various chronic kidney diseases including DKD. EGFR-mediated pathogenesis of DKD is involved in hemodynamic alteration, metabolic disturbance, inflammatory response and parenchymal cellular dysfunction. Therapeutic intervention of this receptor has been available in the oncology setting. Targeting EGFR might also hold a therapeutic potential for DKD. Here we review the functional role of EGFR in the development of DKD, mechanisms involved and the perspective about use of EGFR inhibitors as a treatment for DKD.

## Introduction

Diabetic kidney disease (DKD) is a complication of diabetes mellitus and one of the leading causes of end-stage renal disease (ESRD) worldwide. DKD places a heavy personal burden on the many people world-wide who need hemodialysis and heavy economic burden on health care systems. It is urgent to find ways to slow the progression of DKD. However, its pathogenesis is complex and the mechanism is still poorly understood.

Increasing evidence indicates that various signaling pathways are activated and involved in the pathogenesis of DKD. Among them, the role of epidermal growth factor receptor (EGFR) has been extensively studied (Matrougui 2010; Advani et al., 2011; Zhang et al., 2014; Koya 2015). The EGFR belongs to a family of receptors that harbor tyrosine kinase activity and is composed of four members: EGFR (ErbB1), ErbB2, ErbB3, and ErbB4. They can be activated by several ligands, including epidermal growth factor (EGF), transforming growth factor-α (TGF-α), amphiregulin, heparin-binding EGF-like growth factor (HB-EGF), betacellulin, epigulin and epigen (Higashiyama et al., 2008; Schneider and Wolf 2009; Rayego-Mateos et al., 2018b). Upon ligand binding, the receptors form homodimers or heterodimers, leading to phosphorylation of some specific tyrosine residues in intracellular domains. These residues act as docking sites for initiating activation of multiple intracellular signaling pathways (Forrester et al., 2016).

Activation of EGFR signaling has been implicated in numerous physiological and pathophysiological processes, including embryonic development, cell proliferation, cell survival, and tumorigenesis. In the mammalian kidney, EGFR is widely expressed in glomeruli and proximal tubules, including renal epithelial cells, glomerular endothelial cells, podocytes, tubular cells, mesangial cells and medullary interstitial cells (Gesualdo et al., 1996; Harskamp et al., 2016). In the past decade, many studies have investigated the role of EGFR signaling in the progression of chronic kidney disease (CKD) (Chen et al., 2012; Harskamp et al., 2016; Rayego-Mateos et al., 2018b). In this review, we will discuss the role and the mechanism of EGFR in the development of DKD and consider the potential use of EGFR inhibitors as a treatment of this disease.

### Diabetic Kidney Disease

Chronic hyperglycemia in diabetes mellitus can induce dysfunction of all types of cells in the kidney. DKD is mainly manifested by proteinuria, which varies in several stages, including the silent stage, the microalbuminuria stage (30–300 mg/day) and the macroalbuminuria stage (>300 mg/day) (Papadopoulou-Marketou et al., 2017). Proteinuria occurs along with morphological changes in the glomerulus and interstitium (Qi H. et al., 2017.). From the onset of diabetes mellitus to nearly 5 years, kidney size increases along with an increase in renal plasma flow and hyperfiltration, with thickened glomerular basement membrane and mild or severe mesangial expansion. After 5–10 years, glomerular damage progresses with the occurrence of microalbuminuria and nodular accumulation of mesangial matrix. As glomerulosclerosis advances, extra-glomerular lesions also form. Proteinuria is irreversible at this stage as the glomerular filtration rate (GRF) drops below 60 ml/min/1.73 m^2^ and ultimately reaches end-stage levels below 15 ml/min/1.73 m^2^ (Sulaiman 2019; Han et al., 2017; Kanwar et al., 2011; Papadopoulou-Marketou et al., 2017; Qi C. et al., 2017). During the progression of DKD, mitochondria generate excess reactive oxidative species (ROS) or reactive nitrogen species (RNS), resulting in the activation of several signaling pathways, transcription factors and cytokines, such as TGF-β/smad/MAPK signaling, JAK/STAT signaling, VEGF, EGFR. Activation of these signaling pathways and transcription factors is associated with cell growth, angiogenesis, and apoptosis, leading to DKD ultimately (Kanwar et al., 2011; Magee et al., 2017; Papadopoulou-Marketou et al., 2017; VR et al., 2019).

### Epidermal Growth Factor Receptor Transactivation in Diabetic Kidney Disease

Renal EGFR phosphorylation levels were significantly increased in animal models of diabetes mellitus and in cultured cells treated with high glucose (Konishi and Berk 2003; Saad et al., 2005; Portik-Dobos et al., 2006; Uttarwar et al., 2011; Li R. et al., 2015). EGFR inhibition slowed the progression of DKD, including the improvement of proteinuria and morphologic changes (Wassef et al., 2004; Chen et al., 2012; Zhang et al., 2014). The concentration of EGFR ligands in plasma and kidneys, such as EGF, TGF-α and HB-EGF, was also increased in DKD (Uttarwar et al., 2011; Miyazawa et al., 2013; Perlman et al., 2015). Some reports suggested that connective tissue growth factor (CTGF) is a novel EGFR ligand and that blocking CTGF-mediated profibrotic effects could also be a potential therapeutic option to treat fibrotic renal diseases (Rayego-Mateos et al., 2013; Rayego-Mateos et al., 2018a).

Besides direct activation by its ligands, EGFR transactivation has been recognized as another important mechanism for signal transduction. The process of EGFR transactivation is not mediated through direct ligand binding, but through other second messengers. Several stimuli known to be involved in the pathogenesis of DKD were found capable of transactivating EGFR, such as ROS, TGF-β and PKC. In streptozotocin-induced diabetes and in cultured cells exposed to high glucose, ROS inhibition with superoxide dismutase (SOD) or an NADPH oxidase inhibitor attenuated the upregulation of EGFR phosphorylation (Chen et al., 2015; Sheng et al., 2016). Endothelin-1 (ET-1) mediated activation of endothelin A (ETA) receptor also contributed to EGFR transactivation in diabetic animals (Portik-Dobos et al., 2006). The mechanism by which EGFR transactivation occurs upon stimulation with these active factors remains unclear. A well-accepted hypothesis is that these substances act on their own receptors and then induce release of EGFR ligands (Konishi and Berk 2003; Higashiyama et al., 2008; Chen et al., 2012). EGFR ligands including EGF, HB-EGF and TNF-α are synthesized as precursors anchored on the cell membrane. Upon stimulation, they are released from the membrane in soluble bioactive forms by specific metalloproteases such as ADAM17 (Ohtsu et al., 2006; Uttarwar et al., 2011) (Uttarwar et al., 2011; Li T. et al., 2015; Morgado-Pascual et al., 2015). In diabetes, several second messengers, such as ROS and protein kinases can induce activation of ADAMs, leading to shedding of EGFR ligands. The shed ligands can bind to EGFR in an autocrine or paracrine-dependent manner (Schreier et al., 2014). In addition, Src, a non-receptor tyrosine kinase, can also mediate EGFR transactivation initiated by activation of G-protein-coupled receptors (GPCRs) (Taniguchi et al., 2013; Forrester et al., 2016).

### Epidermal Growth Factor Receptor and the Pathogenesis of Diabetic Kidney Disease

The pathogenesis of DKD is a complex process involving many factors, including hemodynamic alteration, metabolic disturbance, inflammatory response and parenchymal cellular dysfunction (Tung et al., 2018; VR et al., 2019). EGFR transactivation by high glucose causes multicellular dysfunction, which initiates and accelerates kidney injury. Studies have found that EGFR inhibition can reduce kidney size after in STZ treated diabetic mice, without affecting body weight, blood glucose or blood pressure (Wassef et al., 2004). Inhibition of EGFR with erlotinib also markedly reduces albuminuria and renal expression of CTGF, collagen I, collagen IV in diabetic mice (Zhang et al., 2014).

#### Hemodynamic Alteration

Hemodynamic alteration plays an important role in the pathogenesis of DKD. Chronic hyperglycemia induces metabolic alteration and dysfunction in endothelial and vascular smooth muscle cells, leading to vascular dysfunction and hemodynamic alteration in kidneys and other organs (Matrougui 2010; Li T. et al., 2015). Glomerular hemodynamic alterations such as hyperfiltration and hyper-perfusion are found in the early stages of DKD. Hyperfiltration is a result of a decrease in glomerular afferent and efferent arteriolar resistance; dilation of the efferent arteriole is relatively less than dilation of the afferent arteriole, causing a relative increase in glomerular transcapillary hydraulic pressure (Hostetter 2003; Wolf and Ziyadeh 2007; Ziyadeh and Wolf 2008). This facilitates the development of albumin leakage from the glomerular capillary compartment to Bowman’s space. Many factors, especially angiotensin Ⅱ (AngⅡ), have been implicated as important biologically active agents that cause hyperperfusion and hyperfiltration. (Cooper 2001; Wolf 2004; Forbes et al., 2007). Since Ang II can induce transactivation of EGFR, it has been suggested that blockade of EGFR can reduce Ang Ⅱ-mediated hemodynamic alteration (Krishna et al., 2007; Akhtar et al., 2012).

In diabetic animal models, treatment with EGFR inhibitors results in a significant normalization of the altered vasoconstrictor and vasodilator response without effecting blood glucose levels (Benter et al., 2005; Yousif et al., 2005; Benter et al., 2009; Akhtar et al., 2012; Schreier et al., 2014). Mechanistic studies showed that EGFR inhibition mediated vascular response to different stimuli occurs through reduction of ROS generation in mesenteric resistance arteries (Kassan et al., 2015). This may involve the correction of diabetes-induced reduction in nitric oxide synthase (eNOS) activity and nitric oxide (NO) generation in vascular smooth muscle cells (VSMC) (Benter et al., 2015). Despite disturbed vascular response, EGFR also mediates vascular remolding in diabetes. Akhtar and others found that inhibition of EGFR activation results in a remarkable reduction in blood vessels thickening both in intima and media, and attenuates vascular hyper-responsiveness via ERK1/2-ROCK pathway (Palen and Matrougui 2008; Akhtar et al., 2019). Thus, EGFR inhibition could help restore some vascular endothelial functions, independent of glucose lowering, providing considerable therapeutic strategy for vascular protection in DKD.

#### Metabolic Disturbance

Diabetic kidneys are highly sensitive to metabolic alteration. Patients with diabetes mellitus experience chronic hyperglycemia. Glucose was translocated into cells by various transporters including glucose transporter (GLUT)-1, GLUT-4, and sodium-glucose-linked transporters. Excess glucose influx into cells leads to glucose transport along various metabolic pathways, along with the generation of reactive oxygen species (ROS) and advanced glycation end product (AGEs) (Magee et al., 2017). These metabolic derangements induced activation of several signaling pathways related to proliferation and fibrosis, such as the transforming growth factor-β (TGF-β) and the protein kinase C (PKC) signaling pathways (Cooper 2001; Forbes et al., 2007; Kanwar et al., 2011). In addition, researchers found activation of GLUT1 synthesis itself was associated with growth factor upregulation and extracellular matrix secretion (Heilig et al., 2013).

EGFR has been implied in the regulation of the metabolic pathways. In a diabetes model with eNOS knockout, inhibition of EGFR attenuated albuminuria, glomerulosclerosis and tubulointerstitial fibrosis, along with a decreased urinary excretion of F2-isoprostane, a marker of oxidative stress (Portero-Otin et al., 2002; Li et al., 2018). In addition, inhibition of EGFR tyrosine increased glucose tolerance and ameliorated insulin resistance (Li et al., 2018). In STZ-induced diabetes models, EGFR inhibition markedly reduced renal oxidative stress and endoplasmic reticulum stress (ERS), and attenuated renal fibrosis and apoptosis (Xu et al., 2017). In another study, inhibition of EGFR reversed the accumulation of ROS and superoxide levels, probably by improving *p*-eNOS expression and inhibiting Nox4 expression (Wang et al., 2020). Advanced glycation end product receptors (AGERs) are receptors that mediate AGEs-induced toxicity to cells. AGER can interact with EGFR and mediate oxidative species generation, as characterized by H_2_O_2_ formation in mesangial cells and in human embryonic kidney epithelium-like cells (Cai et al., 2006). AGE product precursors could also impair EGFR signaling (Portero-Otin et al., 2002). EGFR activation along with alteration of these metabolic pathways leads to disturbed signaling and mediates kidney injury.

#### Inflammation

Low-grade systemic inflammation seems to play a critical role in the pathogenesis of DKD (Rivero et al., 2009; Matoba et al., 2019; Vasanth et al., 2019). Scurt et al. found that serum markers of inflammation such as CXCL-16, MCP-1, ANGP-2 could predict the onset of microalbminuria in patients with diabetes mellitus type 2 (Scurt et al., 2019). Other studies indicate that serum IL-18 and TNF-α levels were increased in diabetic patients, especially in those with kidney impairment (Moriwaki et al., 2003; Mora and Navarro 2004). In the onset of diabetic mellitus, excess AGEs and ROS, and activation of several signaling pathways, induced transcription of various adhesion molecules and pro-inflammatory cytokines, and mediated macrophage infiltration and the progression of DKD (Matoba et al., 2019). EGFR inhibition decreases renal T-cell infiltration and islet macrophage infiltration in diabetic glomeruli and the interstitium (Li et al., 2018). Aldosterone-induced proinflammatory gene (CCL-2 and CCL-5) expression in cultured tubular epithelial cells was also shown to occur through the ADAM-17/TGF-α/EGFR pathway (Morgado-Pascual et al., 2015). Zhang *et al.* also found that treatment with erlotinib, an EGFR inhibitor, reduced kidney macrophage infiltration and oxidative stress in the tubular interstitium (Zhang et al., 2014). Thus, EGFR may be involved in renal inflammatory responses in DKD.

### Multicellular Dysfunction in Diabetic Kidney Disease

It has been thought that pathologic changes to mesangial cells represent the central feature of glomerulosclerosis in DKD. However, damage to other cell types, including endothelial cells, podocytes, tubular epithelial cells and fibroblasts, also contributes to progression of DKD (Qian et al., 2008; Magee et al., 2017).

#### Mesangial Cell

Mesangial proliferation and expansion is considered the hallmark of DKD. Mesangial cells are mesenchymal in origin, and are easily activated to undergo proliferation and matrix secretion. Activation of growth factors promotes different signaling pathways that mediate proliferation of mesangial cells. Research by Wu and colleagues indicated that high glucose induced collagen production in mesangial cells through EGFR-mediated activation of the PI3K-Akt signaling pathway (Wu et al., 2007; Wu et al., 2009). Another study also indicated that high glucose induced collagen accumulation in mesangial cells through the Src/TACE/HB-EGF signaling pathway (Taniguchi et al., 2013). EGFR transactivation by high glucose does not require PKC, ROS, or AngII, but HB-EGF release is essential for transactivation of EGFR in mesangial cells to induce mesangial cell proliferation and matrix secretion (Uttarwar et al., 2011).

#### Endothelial Cell

Endothelial cells can produce NO and regulate platelet adhesion, immune function, control of volume. Endothelial dysfunction is the inability of vasculature to dilate in response to certain stimuli acting on the endothelium (Goligorsky 2015). It is always associated with a deficiency of eNOS activity and NO release (Rivero et al., 2009; Kolluru et al., 2012; Sharma et al., 2012). Wang et al. applied a novel rhynchophylline analogue, Y396, to study the role of EGFR on endothelial function. They found that Y396 inhibits the tyrosine kinase activity of EGFR by directly targeting EGFR and restores endothelium-dependent vascular relaxation without affecting vascular structure (Wang et al., 2020). This effect of EGFR inhibition may be mediated by downregulation of Nox2 and Nox4 expression and ROS suppression (Galan et al., 2012). Similarly, Belmadani et al. also demonstrated that EGFR activation is elevated and induces resistance artery dysfunction and endothelium-dependent relaxation in diabetic mice without interfering blood pressure (Belmadani et al., 2008).

Another mechanism that regulates vascular dysfunction is the endothelial-to- mesenchymal transition (EndoMT) (Piera-Velazquez et al., 2016; Pardali et al., 2017; Piera-Velazquez and Jimenez 2019). EndoMT is known as endothelial cells transition into a mesenchymal cell type. In 2013, LeBleu et al. employed cell linage tracing and found that approximately 10–15% of myofibroblasts were derived from EndoMT (LeBleu et al., 2013). Since then, much attention has been paid to the EndoMT in DKD, especially the renal interstitial fibrosis (Li et al., 2009; Li and Bertram 2010). The role of EGFR in EndoMT in the kidney was less extensively investigated. In an animal model of cardiac fibrosis, Liu et al. observed that EGFR mediated EndoMT promotes several fibrosis-related events post myocardial infarction, (Liu et al., 2020). These events include acquisition by endothelial cells of a spindle-like shape and the ability to migrate. Although glomerular endothelial mitochondrial dysfunction plays a key role in the pathogenesis of DKD as evidenced by podocyte depletion and proteinuria (Qi H. et al., 2017), the role of EGFR in glomerular endothelial cell pathophysiology has not been well investigated.

#### Podocyte

Podocyte injury is an early event in DKD and is a hallmark of glomerulopathy. Studies have suggested that podocyte injury is associated with the early stage of proteinuria in patients with diabetes (Wolf and Ziyadeh 2007; Bose et al., 2017; Dai et al., 2017). As terminally differentiated cells, podocytes are vulnerable to injury and may not be able to regenerate or repair themselves after injury. They could undergo hypertrophy, epithelial-mesenchymal transition, detachment and apoptosis under certain stimuli, leading to depletion of these cells within the glomerulus, characterized by foot process effacement on biopsy (Han et al., 2017). In DKD, podocytes are involved in the development of glomerular hypertrophy, proteinuria and glomerulosclerosis (Li et al., 2007; Dai et al., 2017; Maestroni and Zerbini 2018) and promote the development of interstitial fibrosis. High concentrations of glucose induce the production of ROS and initiate podocyte apoptosis and podocyte depletion, which may be an early pathological change in DKD (Susztak et al., 2006). EGFR also plays an important role in podocyte injury in DKD. This is evident by the observations that EGFR inhibition led to less podocyte loss in models of diabetic nephropathy while podocyte-specific deletion of EGFR attenuated albuminuria and podocyte loss induced by hyperglycemia (Taniguchi et al., 2013; Li et al., 2018). This may be mediated by activation of TGF-β-SMAD2/3 signaling pathway and enhanced ability of mitochondrial NADPH oxidase to increase ROS production (Chen et al., 2015).

#### Tubular Epithelial Cell

The tubular epithelial cell has been implicated in interstitial fibrosis. Tubular epithelial cells are also vulnerable to pathologic stress due to high glucose levels and can undergo epithelial-mesenchymal-transition and apoptosis (VR et al., 2019). Upon injury, epithelial cells can secrete growth factors and inflammatory cytokines to induce fibroblast activation and renal fibrosis (Sheng and Zhuang 2020). EGFR is highly expressed in proximal tubules. Its transactivation mediates sodium and water transport by regulation of NHE3 and serum glucocorticoid regulated kinase-1 (sgk1) (Panchapakesan et al., 2011). Erlotinib treatment decreased tubular injury and tubulointerstitial fibrosis in db/db mice (Li et al., 2018). EGFR inhibition also attenuated renal tubular epithelial cell proliferation and apoptosis in diabetic rats (Wassef et al., 2004). In addition, erlotinib treatment decreased ER stress and increased autophagy in tubular cells in diabetes (Zhang et al., 2014). The protective effect of some other interventions may be also partly through EGFR. For example, histone deacetylase inhibition can attenuate tubular cell proliferation and early diabetic renal enlargement in response to high glucose by downregulation of EGFR (Gilbert et al., 2011).

EGFR also participated in epithelial-mesenchymal transition (EMT). EMT, a process by which injured renal tubular cells undergo a phenotype change and acquire mesenchymal characteristics, is widely recognized as a critical mediator of fibrogenesis in chronic kidney diseases. EGFR has been thought to mediate EMT. Sustained EGFR activation in the tubule induces epithelial dedifferentiation and cell cycle arrest with an increase in the mesenchymal marker and decreases in the epithelial marker (Overstreet et al., 2017). Administration of CTGF in cultured tubular epithelial cells caused G2/M cell cycle arrest and EMT via EGFR pathways. The cells lost the typical cobblestone pattern and showed a spindle-shaped pattern, along with the elevation of mesenchymal marker and a decrease in epithelial marker. EGFR inhibition attenuated these changes (Rayego-Mateos et al., 2018a). It is possible that the activation of EGFR in diabetic kidney disease may mediate EMT, thus promoting interstitial fibrosis.

### Non-Renal Effects of Epidermal Growth Factor Receptor Inhibitor in Diabetes

Two groups have reported that patients who suffered from non-small-cell lung cancer (NSCLC) experienced an improvement in diabetes after erlotinib (an EGFR inhibitor) treatment (Portero-Otin et al., 2002; Costa and Huberman 2006; Brooks 2012). This improvement may be due to erlotinib-elicited reduction of insulin resistance by inhibition of TNF-α and the T-cell mediated immune response (Brooks 2012). These interesting clinical reports are supported by a striking finding in animal studies showing that erlotinib-treated mice had a relatively slow increase in body weight, a decrease in fasting blood glucose levels, and improved glucose disposition and insulin sensitivity. EGFR inhibition with erlotinib also decreased islet macrophage infiltration and increased autophagy, leading to preservation of pancreatic β-cell function and subsequent improvement of metabolic status. Moreover, EGFR blockade increases circulating levels of the adipokine adiponectin, an adipocyte-derived hormone that has insulin-sensitizing, anti-inflammatory, and kidney-protective effects (Fang et al., 2015; Ding et al., 2016; Li et al., 2018). In addition, treatment with EGFR inhibitor PD153035 reduces low-grade inflammation, macrophage infiltration in adipocytes and improves glucose tolerance and insulin actions (Prada et al., 2009). These studies suggest that EGFR inhibitors may also ameliorate the progression of DKD through improving insulin sensitivity and pancreatic beta cell functions.

### Role of Other Epidermal Growth Factor Receptor Family Members in Diabetic Kidney Disease

Other EGFR tyrosine kinase family members, such as the ErbB2 and ErbB4, may also contribute to the progression of CKD and the pathogenesis of DKD (Zeng et al., 2018a). It relies on heterodimerization with other EGFR family members for signaling. Akhtar et al. investigated the phosphorylation of ErbB2 in diabetes. They found that high glucose exposure enhanced activation of ErbB2, induced vascular dysfunction in VSMCs (Akhtar et al., 2013). ErbB4 expression was increased in the mild fibrotic kidneys, and decreased as fibrosis progressed (Zeng et al., 2018a). ErbB4 suppression significantly attenuated diabetic glomerular injury and albuminuria. Mesangial expansion and sclerosis were reduced with ErbB4 inhibition, as well as STZ-induced podocyte foot process effacement and podocyte loss. TGF-β1 induced MCP-1 expression in podocytes was also suppressed by ErbB4 inhibition (Lee et al., 2017). ErbB4 may also play an important role in glucose homeostasis and lipogenesis. ErbB4 deficiency-related obesity and adipose tissue inflammation may contribute to the development of metabolic syndrome (Zeng et al., 2018b). Some researchers suggest that increased expression of ErbB4 may actually reflect a compensatory effort to prevent development of tubulointerstitial injury (Zeng et al., 2018a).

### Treatment of Diabetes and Diabetic Kidney Disease by Targeting Epidermal Growth Factor Receptor

In the past decades, much attention has been paid on application of tyrosine kinase inhibitors to treat diabetes, including EGFR inhibitors in animal models (Fountas et al., 2015; Malek and Davis 2016). With EGFR inhibitors being extensively used to treat non-small-cell lung cancer (NSCLC), their efficacy in treating CKD and DKD have also been explored in animal models and culture systems. Numerous animal studies and *in vitro* studies have provided evidence that EGFR inhibition could attenuate or prevent development and progression of DKD. This effect may associate with improvement in β cell function and insulin resistance (Li et al., 2018).

Although there are no clinical trials designed for treatment of human DKD by targeting EGFR, there are two case reports about the application of EGFR inhibitor erlotinib in diabetes. In 2006, Costa et al. observed that administration of erlotinib to a lung cancer patient improved his type 2 diabetes (Costa and Huberman 2006). When given chemotherapy with erlotinib 100 mg daily, the patient felt frequent episodes of hypoglycemia, and her fasting glucose level was stabilized as well. After 8 months, her HbA1c had dropped to 6.5% from 8.2%. In another case report, a 73-year-old man with history of metabolic syndrome, CKD and insulin-dependent type 2 diabetes received erlotinib 150 mg daily after being with metastatic NSCLC. Four weeks after starting erlotinib, the patient's insulin requirement began to decline from 90 units daily. After 10 weeks he was off insulin completely. His HbA1c decreased from to 6.6% from 7.4% in six months. At the same time, an abrupt increase in his serum creatinine slowed down (Brooks 2012).

The first EGFR tyrosine kinase inhibitor (TKI) was approved for clinical use in 2003 and was mostly used in patients with non-small-cell lung cancer (NSCLC) carrying EGFR-activating mutations and in patients with breast and pancreatic cancers. Nevertheless, EGFR-TKIs may cause adverse effects. Since EGFR plays a role in epithelial maintenance, the most frequent and severe side effects are dermatological reactions and diarrhea. Other adverse effects include hepatotoxicity, stomatitis, interstitial lung disease, ocular toxicity and hypomagnesaemia (Shah and Shah 2019; Xu et al., 2019; Huang et al., 2020). Most of the data come from patients with cancer. In addition, seven patients were reported in the literature to develop anti-EGFR-induced nephrotic/nephritic syndrome after 2–24 weeks of therapy. All the cases of kidney disease associated with EGFR inhibitor treatment were identified in patients with cancers and shown by the variable and often prolonged time course between drug exposure (2 weeks–6 months) and clinical recognition of kidney injury (Izzedine and Perazella 2017). Since DKD treatment needs a long-term application of drugs, it is anticipated that use of EGFR-TKI in DKD patients would have additional safety concerns. As such, future clinical observations and/or clinical trials are needed to determine the benefit and side effect of EGFR-TKI in those population of patients.

## Conclusion

**FIGURE 1:**
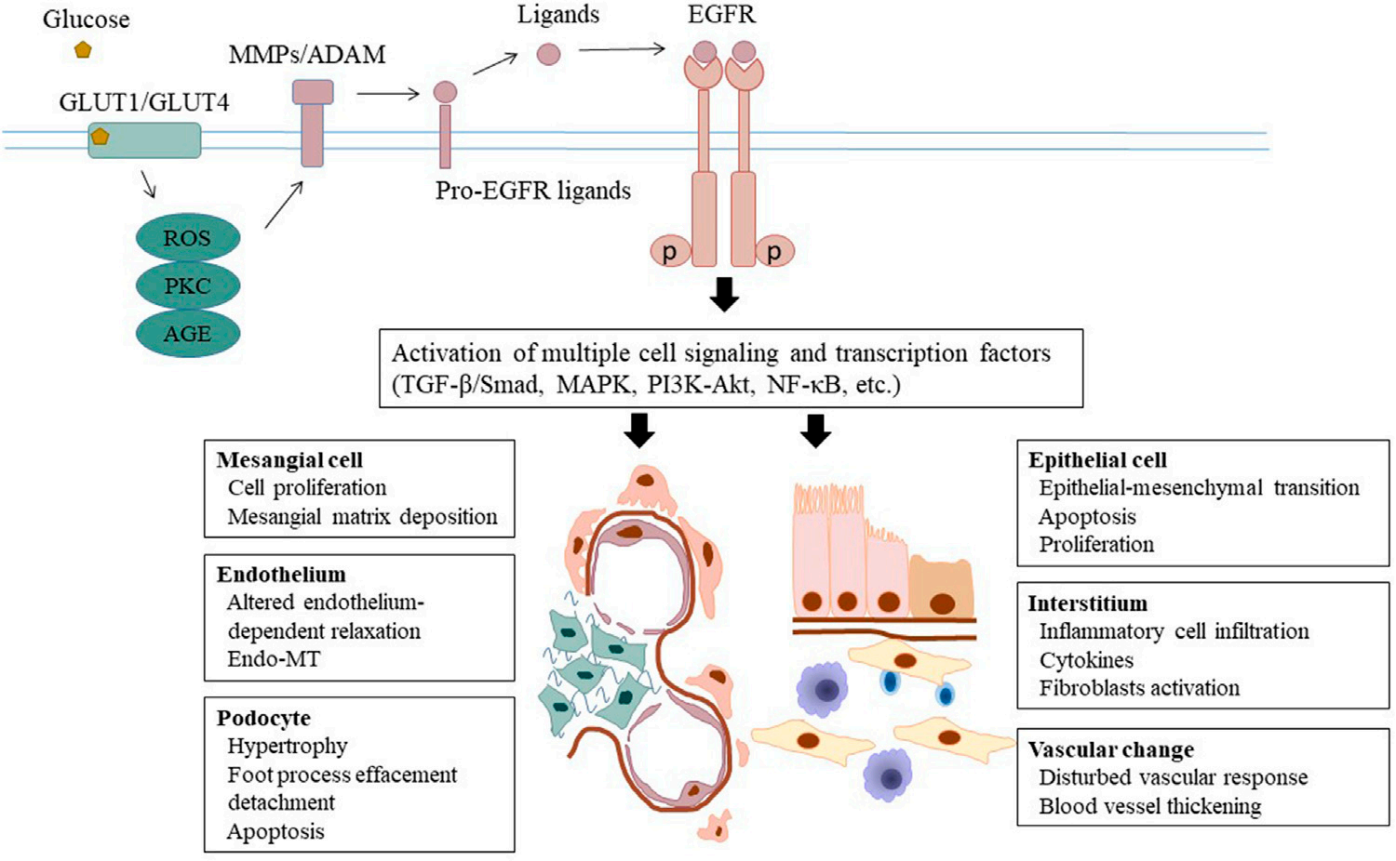
Epidermal growth factor receptor and the pathogenesis of DKD. Under the circumstance of diabetes, glucose triggers activation of ADAMs through several second messengers including ROS and protein kinase C. ADAM-mediated shedding of ligands induces phosphorylation of EGFR and subsequent activation of cell several signaling pathways and transcription factors. As a result, multiple cellular and tissue responses are initiated and implicated in the pathogenesis of DKD progression, including hemodynamic alteration, metabolic disturbance, inflammatory response and parenchymal cellular dysfunction. EGFR, epidermal growth factor receptor; DKD, diabetic kidney disease; ADAM, a disintegrin and metalloprotease; ROS, reactive oxidative species.

Nearly one third of patients with diabetes develop DKD, which in many cases progress to end-stage renal disease and the need for dialysis or kidney transplantation. The underlying mechanisms mediating DKD remain incompletely understood. *In vitro* and *in vivo* studies have demonstrated that EGFR activation can initiate multiple pathological processes leading to DKD, such as hemodynamic and metabolic alterations, chronic inflammation, and multicellular dysfunction (Figure 1 and Table 1). Given the importance of EGFR in mediating the pathogenesis of DKD, much work has gone into studying whether EGR inhibition could slow or stop the development of DKD. EGFR inhibitors have been extensively used to treat various tumors, in particular lung carcinoma. This suggests an interesting possibility that EGFR inhibitors may be repurposed as a treatment for DKD and CKD caused by other etiologies. Nevertheless, beside their benefit effects, long-term use of EGFR inhibitors may result in some adverse effects including kidney problems. Most of side effects of EGFR inhibitors in patients with tumor are tolerable. But it is uncertain whether they are also applicable and tolerable in patients with CKD, in particular DKD. Therefore, clinical trials are needed to determine the efficacy and adverse effects of EGFR inhibitors in patients with DKD.

**TABLE 1:** EGFR and the pathogenesis of DKD.

Factors		Main findings	References
Hemodynamic alternations		Altered vasoconstrictor and vasodilator response	Benter et al. (2015); Akhtar et al. (2019); Palen and Matrougui (2008); Kassan et al. (2015)
Metabolic disturbance		Generation of reactive oxygen species (ROS) and advanced glycation end product (AGEs)	Li et al. (2018); Wang et al. (2020); Cai et al. (2006); Portero-Otin et al. (2002)
Inflammatory response		Inflammatory cell infiltration and proinflammatory cytokine expression	Li et al. (2018); Morgado- Pascual et al. (2015); Zhang et al. (2014)
Parenchymal cellular dysfunction	Mesangial cell	Mesangial cell proliferation and mesangial expansion	Wu et al. (2007); Wu et al. (2009); Taniguchi et al. (2013); Uttarwar et al. (2011)
	Endothelial cell	Altered endothelium-dependent relaxation	Wang et al. (2020); Galan et al. (2012); Belmadani et al. (2008)
		Endothelial-to-mesenchymal transition	Liu et al. (2020)
	Podocyte	Podocyte hypertrophy, detachment and apoptosis	Li et al. (2018); Taniguchi et al (2013); Chen et al. (2015)
	Tubular epithelial cell	Increased ER stress, decreased autophagy; cell proliferation and apoptosis	Li et al. (2018); Wassef et al. (2004); Gilbert et al. (2011)
		EMT	Rayego-Mateos et al. (2013), Morgado-Pascual et al. (2015)

## Author Contributions

LS drafted the article, and GB and SZ edited the manuscript. All the authors reviewed the manuscript and approved is for publication.

## Funding

This work was supported by the National Natural Science Foundation of China (81670623 and 81830021 to SZ, 82000645 to LS), the Branch Grant of National Key Grants of the Ministry of Science and Technology (2018YFA0108802 to SZ) and the US National Institutes of Health (1R01DK113256-01A1 to SZ).

## Conflict of Interest

The authors declare that the research was conducted in the absence of any commercial or financial relationships that could be construed as a potential conflict of interest.
